# Wheelchair skills test questionnaire for manual and powered wheelchair users: arabic translation, adaptation, and validation

**DOI:** 10.3389/fresc.2026.1805871

**Published:** 2026-05-21

**Authors:** Hassan Izzeddin Sarsak

**Affiliations:** Occupational Therapy Program, Batterjee Medical College, Jeddah, Saudi Arabia

**Keywords:** arabic translation, manual wheelchair, occupational therapy, powered wheelchair, reliability, validity, wheelchair skills test questionnaire (WST-Q)

## Abstract

**Background:**

According to the World Health Organization, over 80 million people require wheelchairs for mobility. While these devices are crucial for independence, a lack of training often leads to a deficit in wheelchair-related skills. Standardized assessments like the Wheelchair Skills Test Questionnaire (WST-Q) are vital for establishing baselines, yet no validated Arabic tool previously existed.

**Objective:**

The purpose of this study was to translate and culturally adapt the WST-Q Version 5.0 for manual (WST-Q-M) and power (WST-Q-P) wheelchairs into Arabic and examine their reliability and validity among Arab adult wheelchair users.

**Methods:**

The study followed a rigorous five-stage process: forward translation, synthesis, backward translation, expert committee review, and field testing. The final Arabic versions were administered to wheelchair users across three rehabilitation hospitals. Reliability was assessed via Cronbach's alpha and test-retest Intraclass Correlation Coefficients (ICC), while concurrent validity was measured against the Arabic FEW and WheelCon scales.

**Results:**

The study included 48 participants with an average age of 36.5 years and 7.1 years of wheelchair experience. The sample consisted of 28 males (58%) and 20 females (42%), comprising 26 manual wheelchair users (55%) and 22 power wheelchair users (45%) with diverse medical conditions. Both versions demonstrated excellent internal consistency [manual version: *α* = 0.91, 95% CI [0.86–0.94]; power version: *α* = 0.93, 95% CI [0.89–0.96]]. Test-retest reliability was exceptionally strong, with ICCs ranging from 0.963 to 0.976 across all measured domains. Significant correlations (*p* < 0.01) with FEW and WheelCon scales confirmed strong concurrent validity.

**Conclusions:**

The Arabic WST-Q-M and WST-Q-P are highly reliable and valid tools for clinical and research use. They fill a significant gap, providing Arabic healthcare professionals with a standardized method to guide interventions, prescribe wheelchairs, and track longitudinal progress.

## Introduction

According to the World Health Organization (WHO) estimations, over 80 million persons require a wheelchair to assist their mobility (1). Wheelchairs, both manual and power are crucial for providing mobility, independence, and improved quality of life for people with physical limitations, enabling them to work, study, socialize, and access services, while also offering vital postural support to prevent secondary health issues like pressure sores, improving overall physical and mental well-being, and fostering greater inclusion in society (2, 3). An appropriate wheelchair, according to the WHO, is one that is safe, durable, and properly fits the user's needs and the environmental conditions. It must also be available, affordable, and able to be serviced and maintained within the country where it is used. The WHO also emphasizes a person-centered approach, which includes an individual assessment, fitting, usage and training, and follow-up to ensure the best outcomes for the user (1, 4).

Although wheelchair use may seem straightforward, it can be quite complex, such as in situations where there is a lack of wheelchair training and hence lack of wheelchair-related skills among wheelchair users exist (5–7). Assessment is a key step according to the WHO wheelchair service provision guidelines and the need for validated assessment tools is apparent (1). Standardized assessments, such as those found in the Wheelchair Skills Program (WSP), are used to establish baselines, set goals, and track progress for users and caregivers (8). The program utilizes two primary tools: (1) the Wheelchair Skills Test (WST), an objective performance measure, and the Wheelchair Skills Test Questionnaire (WST-Q), a subjective self-report measure. These measures can be used together and can be complementary. Decisions on which of these assessment methods to use are based on the purpose of the evaluation and, clinically, a combination of methods is typically used (7, 9). The WST-Q serves as a practical alternative when objective testing is impossible and its specific measurement properties for both manual and power wheelchair users have been evaluated (10–15).

The WST-Q is a self-report tool assessing manual and powered wheelchair users' ability (capacity), actual daily use (performance), and confidence in performing essential wheelchair skills, identifying training needs through questions about skills like maneuvering, curb negotiation, and obstacle avoidance, and is a quick, scalable alternative to the hands-on WST. It measures skills in real-world scenarios, assessing capacity (can do), performance (does do), and training goals, providing percentages for overall ability and guiding therapy (13–15). The WST-Q is available in several languages to facilitate international clinical use and research. While the primary versions of the assessment and its manual are maintained in English and French, specific versions have been translated and culturally adapted into Brazilian Portuguese, Colombian Spanish, Turkish, and Norwegian (16–18). These translations allow the WST-Q to serve as a practical, self-reported alternative to objective testing across diverse populations and users are encouraged to refer to the official Wheelchair Skills Program website for the most current updates (16–19).

Up to date, a validated Arabic tool to measure essential wheelchair skills among Arabic-speaking wheelchair users does not exist. Therefore, the purpose of this study was to translate and culturally adapt the WST-Q-M and WST-Q-P into Arabic and examine their reliability and validity in a sample of Arab adult wheelchair users. The importance of this translation is further emphasized by the vast scale of the target population. Arabic is currently spoken by over 400 million people globally, serving as the official language in 22 countries across the Middle East and North Africa (MENA) region. Within these populations, the prevalence of physical disability is significant; however, standardized data on wheelchair use remains fragmented. Recent regional estimations suggest that millions of individuals in the Arab world rely on wheelchairs for daily mobility, yet many face substantial environmental and socio-economic barriers to accessing appropriate devices and rehabilitation services (4). Despite the large number of Arabic-speaking wheelchair users, there has been a historical lack of linguistically and culturally adapted assessment tools. This gap often leads to subjective, non-standardized clinical evaluations, which may not accurately reflect a user's true capacity or training needs (7). By providing a validated Arabic version of the WST-Q, this study addresses an urgent need for an instrument that can be implemented across diverse Arabic-speaking clinical settings to improve service provision and user independence.

## Methods

### Outcome measures

The Wheelchair Skills Test Questionnaire (WST-Q) Version 5.0 is a subjective, self-report assessment tool used to evaluate the wheelchair-use abilities of manual and power wheelchair users, as well as their caregivers. Unlike the objective WST, which requires a physical circuit and observation, the WST-Q can be administered via interview or self-administration in approximately 10 min. For manual wheelchair users, the test typically assesses 33 skills ranging from basic maneuvers (e.g., rolling forward) to advanced tasks (e.g., ascending stairs). For power wheelchair users, the version 5.0 framework includes 25 skills, incorporating power-specific tasks such as operating the controller, changing speed settings, and managing battery charging. Each skill is evaluated across four distinct domains: *Capacity* (what users can do), *Confidence* (how confident and feel they can do), *Performance* (what actually users do in daily life and how often they do it), and Goals (do they want training for this?). Responses are typically recorded on a 4-point ordinal scale (0–3), which allows for the calculation of total percentage scores for each domain. Scores are converted to a 0%–100% range, with higher scores indicating better ability and confidence, often linked to higher life satisfaction and community participation (20–22).

### Translation and cultural adaptation

The study followed a rigorous, multi-stage process based on the ISPOR Task Force “Principles of Good Practice” for Translation and Cultural Adaptation and other established methodological guidelines. This ensured the Arabic versions were linguistically and conceptually equivalent to the original English tools through a standardized approach rather than an *ad hoc* process (23–28).

#### The five-stage process

**Stage 1: Forward Translation:** The English versions were independently translated into Arabic by a native English speaker and an Arabic healthcare provider to create two separate versions.
**Stage 2: Synthesis:** A third independent native speaker merged these two translations into a single synthesized Arabic version.
**Stage 3: Backward Translation:** Two different Arabic translators, who had not seen the original English text, translated the questionnaire back into English.
**Stage 4: Expert Committee Review:** Three expert Arabic occupational therapists reviewed the back-translations against the original English version to ensure all meanings and cultural nuances were maintained.
**Stage 5: Field Testing (Pre-test):** The pre-final version was tested on a sample of 22 participants, consistent with established cross-cultural adaptation guidelines suggesting 15–30 subjects for cognitive debriefing and pilot testing (28). Participants were tested twice to confirm stability with a mean interval of 6.2 days (SD ± 1.4). This timeframe was selected based on methodological standards to be short enough to minimize the likelihood of clinical change while being long enough to reduce recall bias.

#### Final approval

**Figure 1:**
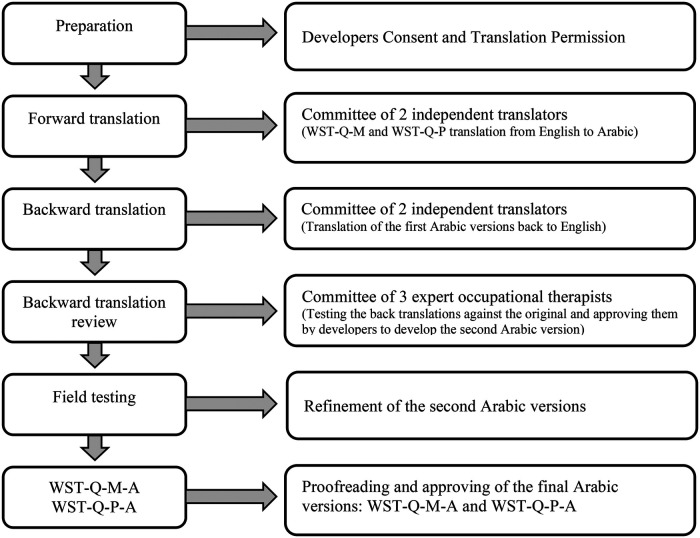
Steps for translating the WST-Q-M and WST-Q-P into arabic language.

Following these stages, the back-translated version was submitted to the original developers for final approval. Because the field testing showed high comprehensibility and no clinical changes between sessions, no items required modification for the final versions (WST-Q-M-A and WST-Q-P-A) (see Figure 1).

### Pre-test (cross-cultural validity)

This pre-final translated version of WST-Q-M and WST-Q-P forms were first applied to 22 patients to evaluate its cross-cultural validity. To avoid bias, each patient was tested twice by the same service provider within one week between repeated administrations to ensure that no clinical change occurred. Based on the results obtained from the field testing, no items were modified. This resulted in the final Arabic version of the WST-Q-M and WST-Q-P forms. The pre-test group (*n* = 22) represented a diverse range of medical conditions, with the most frequent being Cerebral Vascular Accident (36.4%). As detailed in Table 3, the sample also included participants with Above Knee Amputation (22.7%), Spinal Cord Injury (18.2%), Multiple Sclerosis (18.2%), and Traumatic Brain Injury (4.5%). All categorical data were reviewed for consistency, and percentages were rounded to ensure a cumulative total of 100%.

### Participants

According to the validation studies of the tool, a sample size of 30 subjects was required in order to achieve a level of reliability with a Cronbach's *α* > 0.74 and a 95% confidence interval of >0.74 (16–19). The study employed a convenience sampling method, recruiting 48 adult wheelchair users from three specialized tertiary rehabilitation centers in Jeddah, Saudi Arabia. These institutions were selected as they represent primary referral hubs for complex rehabilitation, providing both inpatient and outpatient services for a diverse range of medical conditions including spinal cord injuries, stroke, and traumatic brain injuries. This multi-site approach ensured access to a sample of experienced wheelchair users from varied clinical backgrounds within the region. The sampling frame consisted of all patients currently receiving inpatient or outpatient services at the three specialized tertiary rehabilitation centers in Jeddah, Saudi Arabia, who met the pre-defined eligibility criteria. Recruitment was conducted systematically through two primary methods: direct referral from clinical staff and screening of hospital registries to identify potentially eligible participants. While this non-probability convenience approach facilitated the recruitment of experienced users for reliability testing, we acknowledge it may not fully represent the diverse socioeconomic backgrounds of all Arabic-speaking wheelchair users. Following the field-testing stage, the main validation study recruited a separate cohort of 48 adult wheelchair users from those three different rehabilitation hospitals. To avoid statistical dependence and double-use of data, none of the 22 participants from the pre-test stage were included in this validation sample. The sampling frame consisted of patients currently receiving inpatient or outpatient services at these facilities who met the eligibility criteria. Recruitment was conducted through direct referral from clinical staff and screening of hospital registries. While this non-probability approach facilitated the recruitment of experienced users for reliability testing, it may not fully represent the diverse socioeconomic or regional backgrounds of all Arabic-speaking wheelchair users. A prior power analysis was conducted to determine the required sample size for the test-retest reliability of the Arabic WST-Q. Based on a design with two measurement occasions, a target Intraclass Correlation Coefficient (ICC) of 0.80, an *α* level of 0.05, and a power of 80% to ensure the lower bound of the 95% confidence interval remained above 0.70, the minimum required sample size was calculated as *n* = 39. The recruited sample of *n* = 48 exceeded this threshold, ensuring sufficient empirical justification for the study's reliability targets. To ensure the data was reliable and representative of experienced users, the researchers applied the following specific criteria:

#### Participant eligibility criteria

To ensure the reliability of the data and that the sample represented experienced users, the following inclusion and exclusion criteria were applied (see Table 1). Recruited participants who met the study inclusion criteria were scheduled for two testing sessions and the WST-Q-M-A and WST-Q-P-A forms were administered at both baseline and follow-up by a trained occupational therapist. The participants were patients recruited from the three different rehabilitation hospitals after receiving ethical approvals. All participants were informed about the study and interviewed at the three sites after they gave their consent.

**Table 1:** Inclusion and exclusion criteria for participants.

**Inclusion Criteria**	**Exclusion Criteria**
**Age:** 18 years of age or older.	**Cognitive Impairments:** Individuals with conditions affecting cognitive processing.
**Experience:** Minimum of 6 months using a wheelchair.	**Language Impairments:** Individuals with impairments hindering communication in Arabic.
**Frequency of Use:** Primary mobility device used ≥6 h per day.	
**Communication:** Adequate cognitive status and ability to communicate in Arabic.	

### Reliability

The internal consistency of the WST-Q-M-A and WST-Q-P-A forms were examined by Cronbach's *α* to assess the interrelatedness of the items and the homogeneity of the scale as it was suggested that if a new questionnaire is to be used, its *α* coefficient should be at least 0.7 (29). To evaluate the test–retest reliability, the WST-Q-M-A and WST-Q-P-A forms were administered twice to the participants by the same occupational therapist with a target interval of 7–14 days. For the main validation sample (*n* = 48), the actual mean interval between sessions was 9.4 days (SD ± 2.1). During this period, no wheelchair skills training or clinical interventions were provided to the participants to ensure they remained stable and to prevent bias in the reliability scores. This interval was chosen to be short enough to ensure clinical stability yet long enough to prevent recall of previous answers. The stability of the participants was further confirmed by the field-testing results, which showed no clinical changes between sessions. To measure test–retest reliability, the intraclass correlation coefficient (ICC) was calculated using a two-way mixed-effects model based on a single-measure and absolute agreement (ICC 3,1). Following the guidelines of Koo & Li (2016), the scale was considered stable if the ICC was > 0.70 (30). The 95% confidence intervals for Cronbach's alpha were calculated using the Feldt method via IBM SPSS Statistics version 23.0.

### Validity

Prior to validity testing, the distribution of all scale scores was assessed for normality using the Shapiro–Wilk test. To evaluate concurrent validity, the WST-Q-M-A and WST-Q-P-A forms, the Arabic version of the Functioning Everyday with a Wheelchair (FEW) (31), and the Arabic version of the Wheelchair Use Confidence Scale (WheelCon) (32) were administered together. To evaluate concurrent validity, Pearson correlation coefficients (r) were calculated between the WST-Q-M-A/WST-Q-P-A and the Arabic versions of the FEW and WheelCon scales. To provide a more comprehensive assessment of shared variance and precision, the coefficients of determination (R^2^) and 95% confidence intervals (CI) for each correlation were also reported. The 95% CIs for Pearson correlation coefficients (r) were calculated using Fisher's z-transformation. All statistical analyses were performed using IBM-SPSS version 23.0 (33). To complement the Shapiro–Wilk test and ensure the robustness of our correlation analyses, visual inspection of the data was conducted. Histograms and Q-Q plots were utilized to assess the distribution of scores across all WST-Q-M-A and WST-Q-P-A domains. Additionally, scatterplots with fitted regression lines were generated for all concurrent validity comparisons (WST-Q vs. FEW and WheelCon) to evaluate homoscedasticity and the linearity of the relationships between the scales. To ensure the accuracy of the psychometric evaluations, a complete case analysis protocol was adopted. During the administration of the WST-Q-M-A and WST-Q-P-A, researchers performed an immediate review of the self-report forms to identify any skipped items. Incomplete questionnaires defined as those missing one or more responses in the Capacity, Confidence, or Performance domains were excluded from the final statistical analysis. No imputation methods (such as mean substitution or multiple imputation) were used, as the goal was to evaluate the instrument's performance based on authentic user responses. To assess the stability of the Arabic WST-Q across the diverse sample, secondary subgroup reliability analyses were conducted. Test-retest reliability (ICC 3,1) was stratified by wheelchair type (Manual vs. Power) and gender to ensure consistent instrument performance across these primary demographic divisions.

## Results

### Demographics of subjects (*n* = 48)

A total of 52 adult wheelchair users were initially screened and recruited for the validation cohort. Of these, 4 participants (7.7%) were excluded following data collection due to incomplete responses in the self-report domains (missing one or more responses in Capacity, Confidence, or Performance), precluding their use in the final analysis. This resulted in a final validation sample of *n* = 48 participants, which was entirely distinct from the pre-test group, had an average age of 36.50 years (± 6.25) and an average of 7.1 years of wheelchair experience. The sample consisted of 28 males (58%) and 20 females (42%). The group was nearly evenly split between manual wheelchair users (55%) and power wheelchair users (45%) with diverse medical conditions (see Table 2).

**Table 2:** Study participants' demographics at baseline (*n* = 48).

Demographics	Mean (SD)	*n* (%)
	[range]	
Age (mean, SD)	36.50 (± 6.25)	
[range]	[19.1–44.0]	
Gender		
Male		28 (58)
Female		20 (42)
Type of wheelchair		
Manual		26 (55)
Power		22 (45)
Years using a wheelchair (mean, SD) [range]	7.1 (±5.458) [0.9–12]	
Primary medical condition		
Cerebral Vascular Accident		12 (25.0)
Above Knee Amputation		8 (17.0)
Cardiac Disease		7 (15.0)
Cerebral Palsy		6 (12.5)
Spinal Cord Injury		6 (12.5)
Muscular Dystrophy		3 (6.3)
Multiple Sclerosis		2 (4.1)
Orthopedic Disorder		1 (1.9)
Parkinson Disease		1 (1.9)
Spina Bifida		1 (1.9)
Traumatic Brain Injury		1 (1.9)

### Pre-test (cross-cultural validity)

Cross-cultural validity (field testing) was evaluated on an independent group of 22 participants. The characteristics of these participants are summarized in Table 3. The results were similar to those found using the English original version, and no items were modified to improve comprehensibility and applicability.

**Table 3:** Pretest analysis (*n* = 22).

Demographics	Mean (SD)	*n* (%)
	[range]	
Age (mean, SD)	33.45 (± 7.10)	
[range]	[19.4–45]	
Gender		
Male		12 (55)
Female		10 (45)
Type of wheelchair		
Manual		14 (65)
Power		8 (35)
Primary medical condition		
Cerebral Vascular Accident		8 (36.4)
Above Knee Amputation		5 (22.7)
Cord Injury		4 (18.2)
Multiple Sclerosis		4 (18.2)
Traumatic Brain Injury		1 (4.5)
	Test	Retest
	(mean ± SD)	(mean ± SD)
WST-M-A		
Total Capacity score	81.33 ± 12.44	82.35 ± 14.20
Total Confidence score	75.65 ± 13.14	77.31 ± 14.12
Total Performance score	80.68 ± 12.98	81.32 ± 14.50
WST-P-A		
Total Capacity score	79.57 ± 12.10	78.35 ± 12.15
Total Confidence score	77.41 ± 14.10	78.25 ± 14.25
Total Performance score	79.85 ± 14.10	80.48 ± 12.48

### Reliability

The WST-M-A and WST-P-A were found to have an excellent degree of internal consistency. Internal consistency was excellent for both instruments; the manual version (WST-Q-M-A) yielded an *α* of 0.91 [95% CI (0.86–0.94)] and the power version (WST-Q-P-A) yielded an *α* of 0.93 [95% CI (0.89–0.96)]. These values significantly exceed the standard 0.70 threshold required for clinical scales, indicating high homogeneity among the questionnaire items. The WST-M-A and WST-P-A demonstrated high item-total correlations across all items, further supporting the scale's internal homogeneity (see Table 4).

**Table 4:** Test-retest analysis (*n* = 48).

Outcome measure	Test (mean ± SD)	Retest (mean ± SD)	ICC (3,1) [95% CI]
WST-M-A			
Capacity	81.44 ± 13.80	82.45 ± 12.11	0.963 [0.944; 0.977]
Confidence	83.76 ± 14.80	83.45 ± 14.43	0.965 [0.954; 0.979]
Performance	83.89 ± 14.74	84.23 ± 12.33	0.966 [0.898; 0.978]
WST-P-A			
Capacity	81.65 ± 12.34	82.45 ± 13.54	0.972 [0.933; 0.975]
Confidence	82.11 ± 12.67	83.76 ± 13.64	0.974 [0.931; 0.979]
Performance	84.45 ± 12.45	85.74 ± 13.36	0.976 [0.921; 0.981]

### Subgroup reliability analysis

To evaluate the stability of the Arabic WST-Q across different clinical and demographic profiles, secondary reliability analyses were performed. The exceptionally strong test-retest reliability observed in the aggregate sample was consistently maintained when stratified by primary subgroups. *Wheelchair Type:* Both the manual (WST-Q-M-A) and power (WST-Q-P-A) versions demonstrated high stability, with ICC values of 0.963 and 0.972 for the Capacity domain, respectively. *Gender:* Reliability remained robust across genders, with the Capacity domain showing an ICC of 0.968 for males (*n* = 28) and 0.965 for females (*n* = 20). *Clinical Diversity:* Despite the sample's heterogeneity, which included 11 different medical conditions such as CVA (25%) and SCI (12.5%), the narrow 95% confidence intervals across these stratified analyses indicate that the instrument's performance is not significantly skewed by the user's specific diagnosis. *Domain Consistency:* The Performance and Confidence domains also showed high consistency within these subgroups, mirroring the aggregate internal consistency scores of 0.91 for manual and 0.93 for power versions (see Table 5). This consistency across diverse user profiles confirms that the Arabic WST-Q is a versatile tool suitable for a broad range of Arabic-speaking wheelchair users in various clinical settings.

**Table 5:** Stratified reliability analysis by subgroup.

Subgroup	N	WST-Q Domain	ICC (3,1)	95% CI
Wheelchair Type				
Manual (WST-M-A)	26	Capacity	0.963	[0.944, 0.977]
Power (WST-P-A)	22	Capacity	0.972	[0.933, 0.975]
Gender				
Male	28	Capacity (Combined)	0.968	[0.942, 0.981]
Female	20	Capacity (Combined)	0.965	[0.938, 0.979]

### Validity

**Figure 2:**
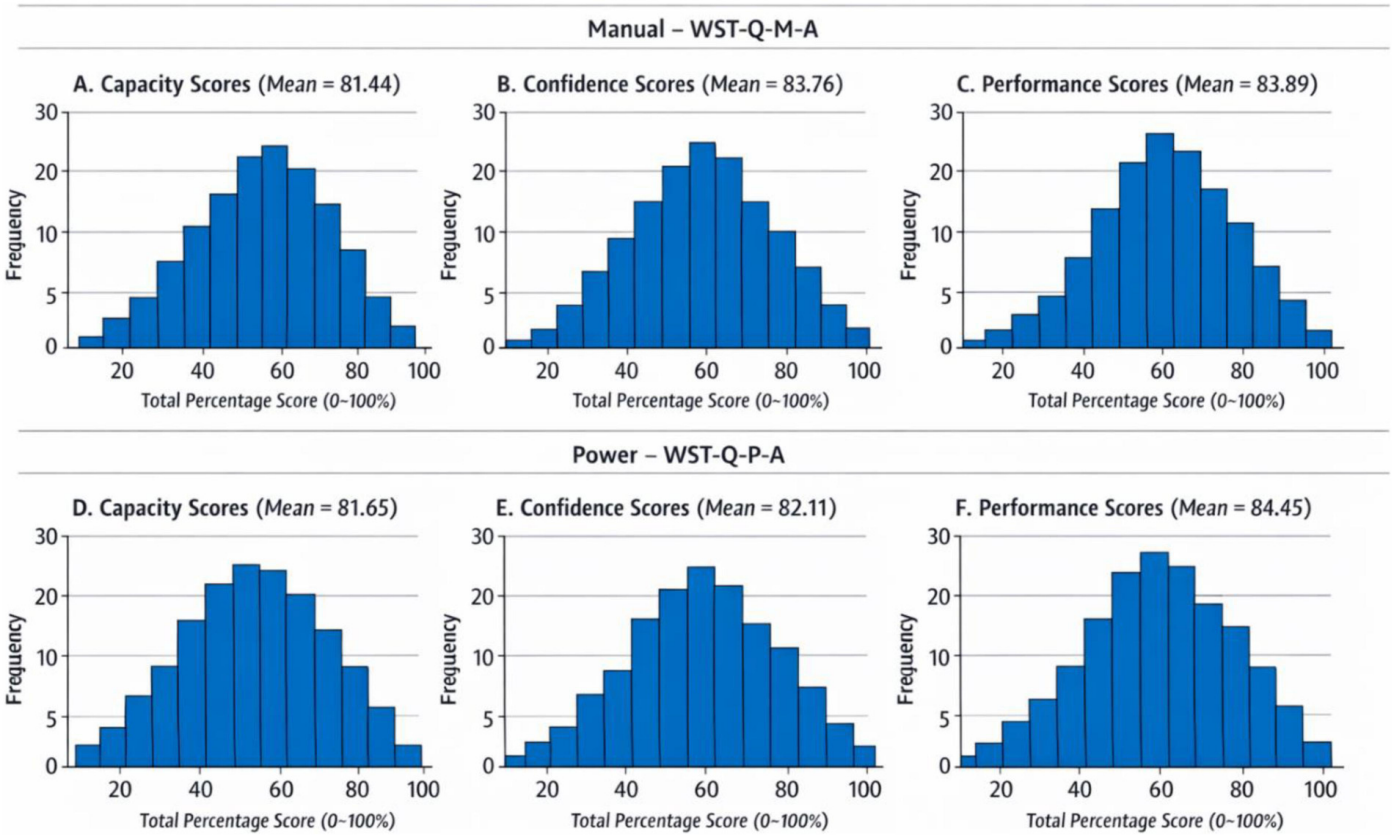
Distribution of percentage scores for the arabic WST-Q-M and WST-Q-P domains.

**Figure 3:**
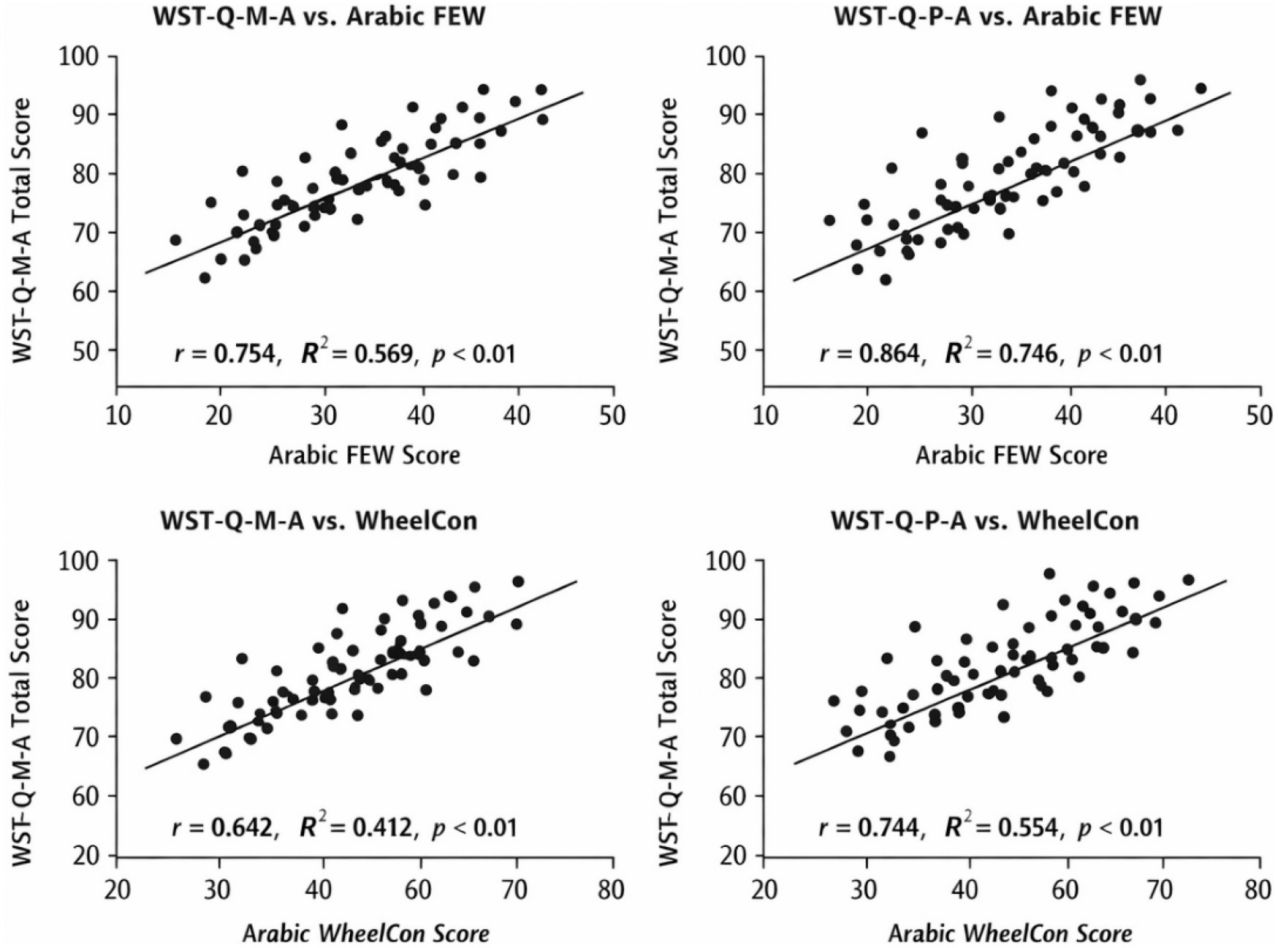
Scatterplots showing concurrent validity between the arabic WST-Q versions and established measures (FEW and wheelCon).

Visual inspection of histograms for the manual version (WST-Q-M-A) generally showed a normal distribution of scores, which was corroborated by the Shapiro–Wilk test. However, histograms for the power version (WST-Q-P-A) exhibited moderate left-skewness, particularly in the Capacity and Performance domains, justifying the use of Spearman's rho (*ρ*) alongside Pearson's r to ensure robust validity measurement. Scatterplots for concurrent validity demonstrated a clear linear relationship between the Arabic WST-Q versions and the criterion scales (FEW and WheelCon). The scatterplots revealed consistent shared variance across the spectrum of wheelchair experience levels, with no significant outliers or non-linear patterns detected, supporting the high coefficients of determination (R^2^) reported in Table 6 (see Figures 2, 3). Normality testing via Shapiro–Wilk indicated that while many domains were normally distributed, some scores (particularly in the power version) showed non-normal distributions. Spearman's rho (*ρ*) was consistent with Pearson's coefficients (r), with all values indicating strong concurrent validity. Specifically, the WST-Q-M-A correlated with the FEW at 0.751 (*ρ*) and with the WheelCon at 0.638 (*ρ*)″, while the power version showed even stronger correlations of 0.861 and 0.742, respectively (*p* < 0.01 for all) (see Table 6). Analysis of the score distributions for both the WST-Q-M-A and WST-Q-P-A revealed no significant floor or ceiling effects. Floor and ceiling effects were defined as more than 15% of participants achieving the lowest (0%–10%) or highest (90%–100%) possible scores, respectively. *WST-Q-M-A:* For the manual version, the proportion of participants scoring >90% was 4.2% (*n* = 2) for Capacity, 6.3% (*n* = 3) for Confidence, and 6.3% (*n* = 3) for Performance. No participants scored <10% in any manual domain. *WST-Q-P-A:* For the power version, scores >90% were reported by 9.1% (*n* = 2) for Capacity, 4.5% (*n* = 1) for Confidence, and 9.1% (*n* = 2) for Performance. Similarly, no participants scored <10% in the power domains. The visual inspection of histograms in Figure 2 confirmed that scores were well-distributed across the 0%–100% range without excessive clustering at the extremes.

**Table 6:** Concurrent validity analysis (Pearson and spearman's coefficients).

Measure	Scale	Pearson r	Spearman *ρ*	R2	95% CI	p-value
WST-Q-M-A	FEW	0.754	0.751	0.569	[0.52, 0.89]	< 0.01
	WheelCon	0.642	0.638	0.412	[0.34, 0.81]	< 0.01
WST-Q-P-A	FEW	0.864	0.861	0.746	[0.69, 0.94]	< 0.01
	WheelCon	0.744	0.742	0.554	[0.46, 0.89]	< 0.01

*p* < 0.01 (two-tailed).

### Key observations

#### High stability

Both versions demonstrated “excellent” internal consistency and “exceptionally strong” test-retest reliability across all domains. The test-retest reliability was evaluated with an average inter-session period of 9.4 ± 2.1 days for the main cohort, ensuring clinical stability while minimizing recall bias. Also, field testing was conducted with a mean interval of 6.2 ± 1.4 days between repeated administrations.

#### Stronger validity in power users

The correlation with the “gold standard” measures (FEW and WheelCon) was slightly higher for the power wheelchair version than for the manual version.

#### Clinical meaningfulness

The high magnitude of the reliability coefficients (*α* > 0.90 and ICC > 0.96) and the substantial shared variance (R^2^ up to 74.6%) with established measures like the FEW confirm that the Arabic WST-Q is not only statistically stable but also clinically relevant for assessing wheelchair skills. Here is the comparison of the reliability and validity scores for the Arabic versions of the manual and power wheelchair questionnaires (see Table 7).

**Table 7:** Reliability and validity comparison.

Metric	Manual Version (WST-Q-M-A)	Power Version (WST-Q-P-A)
Internal Consistency (Cronbach's alpha)	0.91	0.93
Test-Retest Reliability (ICC)—Capacity	0.963	0.972
Test-Retest Reliability (ICC)—Confidence	0.965	0.974
Test-Retest Reliability (ICC)—Performance	0.966	0.976
Concurrent Validity (FEW Correlation)	0.754	0.864
Concurrent Validity (WheelCon Correlation)	0.642	0.744

## Discussion

The primary objective of this research was the translation and cultural adaptation of the Wheelchair Skills Test Questionnaire (WST-Q) Version 5.0 for both manual (WST-Q-M) and powered (WST-Q-P) wheelchair users into Arabic. Before this study, clinicians in the Arab world lacked a validated, language-specific instrument to measure essential wheelchair skills across the domains of capacity, performance, and confidence. By developing the WST-Q-M-A and WST-Q-P-A, this work provides healthcare professionals with a standardized tool to guide clinical interventions and track longitudinal progress.

Our results indicate that these Arabic versions are highly reliable and valid for clinical and research use. The statistical power was robust, as the final sample size (*n* = 48) surpassed the sample size requirement (*n* = 39) needed to confirm stable ICC thresholds across all domains. Internal consistency was excellent, with Cronbach's *α* values reaching 0.91 [95% CI (0.86–0.94)] for the manual version and 0.93 [95% CI (0.89–0.96)] for the power version, both well above the standard 0.70 threshold for clinical scales (34). Furthermore, test-retest reliability was exceptionally strong, with coefficients ranging from 0.963 to 0.976 across all measured domains, indicating excellent stability over the average 9.4-day retest period. The consistency of these intervals across participants helps ensure that the high ICC values (0.963–0.976) accurately reflect the stability of the Arabic WST-Q rather than variance introduced by fluctuating inter-session timings. The slightly wider and asymmetric confidence interval observed in the Performance domain of the manual version (WST-M-A) warrants consideration. This increased variance may be attributed to the inherent nature of the “Performance” construct, which captures actual daily usage rather than peak capability. Environmental barriers or changes in daily activity levels between the two measurement occasions may have introduced greater item-level variance compared to the more stable “Capacity” and “Confidence” domains. Despite this, the lower bound of 0.898 remains well above the 0.70 threshold for stability, confirming the tool's reliability for clinical use. While statistical significance was achieved across all measures (*p* < 0.01), the clinical utility of the WST-Q-M-A and WST-Q-P-A is best reflected in the strength of their reliability coefficients. The exceptionally strong test-retest reliability (ICC range 0.963–0.976) ensures that observed changes in scores over time likely reflect true clinical progress rather than measurement error (35). While test-retest reliability was exceptionally strong across all domains, the WST-M-A Performance domain exhibited a wider 95% confidence interval (0.898–0.978) compared to other domains. This indicates a slightly higher degree of variability in self-reported daily performance among manual wheelchair users between the two testing sessions.

These statistics suggest that the translation process preserved the conceptual integrity of the original English framework while remaining stable for use with Arabic-speaking populations.

When compared to existing literature, these findings mirror successful validation efforts in other languages, such as Brazilian Portuguese, Colombian Spanish, and Turkish (16–19). The concurrent validity of the Arabic versions was established through significant correlations with the Arabic Functioning Everyday with a Wheelchair (FEW) and the Wheelchair Use Confidence Scale (WheelCon). Specifically, the WST-Q-M-A correlated at 0.754 with the FEW and 0.642 with the WheelCon, while the power version showed even stronger correlations of 0.864 and 0.744, respectively. To further assess the depth of these relationships, the coefficients of determination (R^2^) were analyzed. For the manual version, shared variance reached up to 56.9% (R^2^ = 0.569) with the FEW, while the power version demonstrated a highly robust shared variance of 74.6% (R^2^ = 0.746). These high R^2^ values, supported by narrow 95% confidence intervals, provide stronger evidence of criterion validity than correlation coefficients alone. This aligns with previous evidence suggesting the WST-Q is a robust alternative to objective performance testing (9, 12, 13, 22, 23). The absence of significant floor and ceiling effects reinforces the validity of the Arabic WST-Q versions. Because participants represented a wide range of ability levels from newer users to those with over 12 years of experience, the instruments demonstrated a sufficient range of scores to capture variance in wheelchair skills. This lack of restriction-of-range ensures that the high concurrent validity correlations (reaching up to *r* = 0.864 for power users) are robust and not artificially deflated or inflated by score clustering. The high coefficients of determination (R^2^ up to 74.6%) further suggest that the Arabic WST-Q is sensitive enough to differentiate between varying levels of mobility capacity and confidence within the Arabic-speaking clinical population.

### Clinical implications for healthcare professionals

The Arabic versions of the Wheelchair Skills Test Questionnaire (WST-Q) Version 5.0 are now available for Arabic healthcare professionals and can be used with Arabic-speaking manual and power wheelchair users in clinical practice and research. The Arabic WST-Q can be used to measure Arabic-speaking manual and powered wheelchair users' ability (capacity), actual daily use (performance), and confidence in performing essential wheelchair skills. This study is the first to fill the gap in identifying areas of wheelchair-related skills for Arabic-speaking adult manual and power wheelchair users. This will help clinicians make informed decisions when prescribing wheelchairs, measuring wheelchair skills, training wheelchair users, guide interventions, and measuring change over time.

The validation of the WST-Q-M-A and WST-Q-P-A allows therapists to move beyond general observations and use a standardized, evidence-based approach to wheelchair service provision. These results can be integrated into a hospital or rehabilitation setting by (1) informed prescription and fitting; clinicians can use the capacity scores to determine if a specific wheelchair type safely fits the user's physical needs and environmental conditions, (2) targeted intervention planning; by identifying specific skill deficits (e.g., curb negotiation or battery management), therapists can guide interventions to focus on the areas where the user lacks confidence or ability, (3) goal setting and training; the “Goals” domain of the questionnaire allows the user to identify which skills they want to prioritize, ensuring a person-centered approach to wheelchair training programs, (4) measuring progress over time; because the tool is reliable and stable (high ICC), it can be used at baseline, during discharge, and at follow-up to objectively measure change and the effectiveness of rehabilitation, and (5) resource efficiency; since the WST-Q can be administered via interview in approximately 10 min and does not require a physical obstacle circuit, it serves as a scalable alternative to hands-on testing in busy clinical environments.

### Limitations and future direction

Despite these strengths, certain limitations must be acknowledged. The use of convenience sampling from tertiary centers introduces a degree of selection bias that remains partially unquantified. Patients in these settings may have different skill levels or medical complexities compared to wheelchair users in the community who do not seek regular clinical services. Furthermore, while our attrition rate was low (7.7%), the use of complete case analysis may introduce a minor degree of attrition bias, as users with more difficulty completing the questionnaire were excluded from the final psychometric evaluation. Participants recruited from rehabilitation hospitals may possess different skill levels, access to training, or medical complexities compared to wheelchair users in the community who do not seek regular clinical services. From a statistical perspective, while the sample size was sufficient to achieve a Cronbach's *α* > 0.74, the lack of a probability-based sample means the findings may not be generalizable on a regional or national level. The results primarily reflect the psychometric stability of the tool within a clinical population rather than the average skill levels of the general Arabic-speaking population. Future research should utilize stratified random sampling across multiple Arabic-speaking countries to account for linguistic variations and diverse environmental factors. While the sample size was statistically justified by our power analysis and sufficient for validation, the 48 participants may not fully capture the diverse linguistic and environmental variations found across different Arabic-speaking regions. The current validation study was conducted exclusively with participants in Saudi Arabia. While the translation process prioritized Modern Standard Arabic to ensure broad conceptual equivalence, the considerable diversity of Arabic dialects and varying environmental infrastructures across the Gulf, Levant, and North Africa may influence how certain items are perceived or reported. Consequently, these findings should be viewed as an essential first step in the regional validation of the WST-Q. Future research should utilize stratified random sampling across multiple Arabic-speaking countries to account for these linguistic variations and diverse environmental factors. Such multicenter studies will be crucial to confirm the tool's cultural validity and clinical utility across the entire Arabic-speaking population. Furthermore, while the use of complete case analysis ensured data purity, it may introduce a degree of attrition bias. Future large-scale studies might explore the use of modern imputation techniques to better understand the patterns of missingness, particularly if certain advanced wheelchair skills are consistently left blank by specific user subgroups. Additionally, because the WST-Q is a self-report measure, it reflects the user's perception of their skill, which can occasionally differ from objective physical performance. In addition, the increased variability noted in the manual performance domain suggests that self-reported daily use may be more susceptible to environmental or situational fluctuations than capacity-based assessments. The study population was limited to adults, meaning these findings cannot yet be generalized to pediatric wheelchair users without further investigation. The high magnitude of reliability coefficients across both manual (ICC = 0.963) and power (ICC = 0.972) versions suggests that the Arabic WST-Q is robust against the clinical heterogeneity of the population. While the sample included 11 different primary medical conditions, the consistently narrow confidence intervals across domains indicate that the questionnaire maintains its stability regardless of the underlying pathology. We acknowledge that the sample size was insufficient to provide a reliable ICC for individual rare diagnoses (e.g., Multiple Sclerosis or Parkinson's Disease), which remains a direction for future large-scale multicenter studies. Nonetheless, the current findings support the clinical utility of the WST-Q-M-A and WST-Q-P-A as versatile tools for the broader Arabic-speaking rehabilitation community. Future research should focus on extending this validation to the geriatric population to ensure the tool's utility across different age groups. Additionally, longitudinal studies could evaluate the instrument's responsiveness to specific rehabilitation interventions and wheelchair training programs. By integrating these Arabic versions into standard practice, clinicians can improve the precision of wheelchair prescriptions and training, ultimately fostering greater independence and community participation for wheelchair users in the region.

## Conclusions

This study successfully translated and culturally adapted the Wheelchair Skills Test Questionnaire (WST-Q) Version 5.0 into Arabic for both manual and power wheelchair users, filling a significant gap in validated assessment tools for Arabic-speaking populations. Through a rigorous forward and backward translation process involving expert committees, the research established that the Arabic versions (WST-Q-M-A and WST-Q-P-A) possess excellent internal consistency and high test-retest reliability. The tools demonstrated strong concurrent validity through significant correlations with the FEW and WheelCon scales, proving they are reliable and valid measures of a user's capacity, performance, and confidence. These findings provide Arabic healthcare professionals with a standardized method to guide clinical interventions, prescribe appropriate wheelchairs, and track longitudinal progress, ultimately fostering greater independence for wheelchair users in the region.

## Data Availability

The raw data supporting the conclusions of this article will be made available by the authors, without undue reservation.
